# Chronic Myeloid Leukemia Prognosis and Therapy: Criticisms and Perspectives

**DOI:** 10.3390/jcm9061709

**Published:** 2020-06-02

**Authors:** Domenico Russo, José Valentín Garcia-Gutierrez, Simona Soverini, Michele Baccarani

**Affiliations:** 1 Chair of Hematology, Unit of Blood Diseases and Bone Marrow Transplantation, Department of Clinical and Experimental Sciences, University of Brescia, ASST-Spedali Civili, 25100 Brescia, Italy; 2 Servicio de Hematología. Hospital Universitario Ramón y Cajal, IRYCIS, 28034 Madrid, Spain; jvalentingg@gmail.com; 3 Institute of Hematology “Lorenzo e Ariosto Seràgnoli”, University of Bologna, 40138 Bologna, Italy; simona.soverini@unibo.it (S.S.); michele.baccarani@unibo.it (M.B.)

**Keywords:** prognosis, therapy guidelines, tyrosine kinase inhibitor, MRD monitoring, quantitative PCR, digital PCR, NGS mutation, treatment free remission, treatment de-escalation

## Abstract

Ph+ chronic myeloid leukemia (CML) is a clonal myeloproliferative disease whose clinical course is characterized by progression disease from the early chronic phase (CP) to the fatal blastic phase (BP). This programmed course is closely related to the translocation t(9;22)(q22;q11) and the resulting BCR-ABL1 fusion protein (p210) that drives the leukemic transformation of hematopoietic stem cells. Therefore, the cure of CML can only pass through the abrogation of the Ph+ clone. Allogeneic stem cell transplantation (allo-SCT) and interferon-alpha (IFNα) have been proven to reduce the Ph+ clone in a limited proportion of CML population and this translated in a lower rate of progression to BP and in a significant prolongation of survival. Tyrosine-kinase inhibitors (TKIs), lastly introduced in 2000, by preventing the disease blastic transformation and significantly prolonging the survival in up to 90% of the patient population, radically changed the fate of CML. The current therapy with TKIs induces a chronicization of the disease but several criticisms still persist, and the most relevant one is the sustainability of long-term therapy with TKIs in terms of compliance, toxicity and costs. The perspectives concern the optimization of therapy according to the age, the risk of disease, the potency and the safety profiles of the TKIs. The prolongation of survival is the most important end point which should be guaranteed to all patients. The treatment free remission (TFR) is the new goal that we would like to give to an increasing number of patients. The cure remains the main objective of CML therapy.

Philadelphia-positive (Ph+) chronic myeloid leukemia (CML) is a clonal myeloproliferative disease marked by chromosome translocation t(9;22) (q22;q11) that leads to the *BCR-ABL1* fusion gene. The resulting BCR-ABL1 fusion protein (p210), is a constitutively activated tyrosine kinase that drives the leukemic transformation of hematopoietic stem cells, and induces the progression of the disease from the early chronic phase (CP) to the blastic phase (BP) which fatally close the course of the disease [1,2,3,4].

Over the past century, the treatment of CML has moved from observation alone to chemotherapy (mainly busulfan and hydroxurea) and from allogeneic stem cell transplantation (allo-SCT) or interferon-alpha (IFNα) to tyrosine-kinase inhibitors (TKIs), lastly introduced in 2000 [5].

The main teaching of the countless clinical and biological studies conducted over all these years is that the cure of CML can only pass through the abrogation of the Ph+ clone, which can be detected and monitored by cytogenetics (chromosome banding analysis (CBA), or fluorescent in-situ hybridization (FISH)) or by real time quantitative reverse transcription polymerase chain reaction (RT-qPCR) [6,7,8].

At the turn between the 1980s and 1990s, allo-SCT and IFNα proved to reduce the Ph+ clone down to achievement of complete cytogenetic response (CCyR) which means the disappearance of Ph+ metaphases by CBA, although this occurred in a minority of patients. This translated to a lower rate of progression to BP and in a significant prolongation of survival. However, the benefit was limited to no more than 10% of young (<50 years) CML patients suitable to be allo-transplanted in CP and to less than 10%–15% of IFN-treated patients achieving CCyR [5,6,9,10].

The evidence of a potential eradication of the Ph+ clone was a great success, but the overall benefit for CML patients was limited. However, by demonstrating the effectiveness of the targeting BCR-ABL1 clone, the route towards the cure of the disease had been traced.

In the same years, many studies on CML prognosis were done. They were equally relevant and have had the merit of teaching us other important things.

The risk of disease progression is not the same in all newly diagnosed patients and intensification of therapy through transplantation or testing new therapeutic approaches had to be primarily reserved for patients with high-risk disease or negative prognostic factors, able to predict earlier blastic transformation [11].

The Sokal score, generated in the 1980s, still represents the reference for defining the risk of disease progression at diagnosis. The Sokal score has been paired, but not replaced, by the Euro score generated and adapted for patients candidated to receive IFNα therapy and, more recently, by the EUTOS score for patients receiving TKIs. Although it is based on simple clinical and hematologic parameters (age, spleen size, platelet and blast cells count), it is still used as it remains a fundamental tool for planning the therapeutic strategy [12,13,14,15].

In recent decades, a high number of biological studies have been done to elucidate the molecular mechanisms of CML pathogenesis and progression [16,17,18]. The results of these studies were fundamental to understand how and why the p210 tyrosine kinase protein is able to drive the leukemic transformation of Ph+ hematopoietic progenitors by altering cell proliferation, apoptosis, adhesion and inducing genomic instability [1,2]. Now, we can say that these studies paved the way for the current target therapy [19].

The advent of TKIs in the 2000s radically changed the fate of CML, since imatinib (IM), before, and nilotinib (NIL), dasatinib (DAS) or bosutinib (BOS), after, showed to be able to prevent the disease blastic transformation and significantly prolong the survival [20,21,22,23]. Achieving such a goal in up to 90% of the patient population treated first line with TKIs could mean that cure of CML have been finally achieved, but this is not completely true.

Several criticisms persist, and the most relevant one is the sustainability of long-term therapy with TKIs in terms of compliance, toxicity and costs [19,24].

Indeed, to prolong survival, all patients should take any TKI, at the standard dose, daily and lifelong. The adherence and tolerance to chronic treatment, the onset of late and unexpected side effects, the worsening of quality of life, and the high costs of therapy are still open questions.

Since the median age of CML is 60 years, approximately 50% of patients are younger and have a life expectancy of 25–30 years [19]. Thus, can TKI treatment be continued for such a long period of time? The other 50% of CML patients are older than 60 years [25]. Considering that tolerance and adherence to TKI therapy progressively decrease by time and by age, do low compliance and treatment adherence compromise therapy effectiveness in the elderly?

These questions are clinically and socially relevant, because it is known that the incidence of CML progresses with age and, in the next years, the prevalence of CML in the elderly population is expected to increase two- or three-fold. Therefore, for different reasons, it is clear that a long-term therapy with TKIs is not easily sustainable for the great majority of patients.

Furthermore, considering that the cost of TKI therapy/year may range from 10,000 to 42,000 Euros, we can easily understand the huge amount of money that the National Healthcare Systems have to sustain for many years [24].

Unfortunately, neither IM nor the more potent second-generation TKIs (e.g., NIL or DAS) are able to eradicate the Ph+ leukemic stem cells (Ph+ LSC) and allow a “biological cure” of the disease [26,27,28]. However, the observation that a limited number of patients can achieve a level of molecular minimal residual disease so low that the treatment can successfully be discontinued has opened to the possibility of obtaining an “operational cure”, and considering the treatment free remission (TFR) as a goal of CML therapy [29].

The most recent recommendations on the CML management highlight to achieve the treatment discontinuation (TD) and maintain TFR but, at the same time, they do not clarify if TFR is a cost-effective strategy and right for all CML patients [5].

In several trials, hundreds of patients aged up to 75 years receiving IM, NIL or DAS have been selected for TD after achieving a deep (≥MR4.0, defined as 4-log decrease in BCR-ABL1 transcript levels from the standardized baseline) and durable (2 or 3 years) molecular response (DMR). Out of these, 40%–50% maintain TFR, while the remaining 50%–60% of patients losing major molecular response (MMR or MR3.0) can safely regain the previous DMR by reassuming the daily therapy, without any risk of disease progression [30].

However, the current TKI discontinuation strategies are still too far from being considered optimal because the definitions of “deep” and “durable” MR are uncertain and inaccurate and the selection of patients is thus not reliable.

Assessing DMR through the molecular measurement of BCR-ABL1 transcript levels in the peripheral blood by RT-qPCR is necessary for addressing the patient to TKI discontinuation but, unexpectedly, a tight correlation between the depth and duration of DMR, and the rate of TFR maintenance does not exist. The reason might be sought in the intrinsic limitations of RT-qPCR, mainly consisting of lack of precision in the quantification of the low levels of the target (BCR-ABL1 transcript) [31,32]. Thus, the great majority of patients who undergo the treatment discontinuation are preferably selected among those with undetectable levels of *BCR-ABL1* transcript by RT-qPCR [33,34]. Despite this restrictive selection, 50%–60% of patients with undetectable MR by RT-qPCR lose MMR within the first year of discontinuation [30,35,36]. Therefore, the benefit of TD and TFR policy is restricted to no more than 15%–25% of all CML patient population. This rate is quite similar to the proportion of patients who have benefited from transplantation or IFNα in the past.

Even if no more than 50% of patients achieving a DMR (less than 25% of the overall CML population) maintain the TFR after TKI discontinuation, this policy has become common clinical practice during the last years [37,38,39,40].

At present, several trials are challenging a second TFR in those patients who failed the first one, by using different and more potent TKIs, or experimental combinations including new drugs with different mechanisms of action [41,42]. These exploratory studies aimed to get a second TFR suggest that the first line TFR strategy may be very fascinating but largely bankrupt. Consequently, the great majority of CML patients (>60%–70%), at present, do not have any valid alternative other than to continue the standard TKI treatment, daily and lifelong.

Looking at the patients undergoing TKI discontinuation, there is a tendency to reserve the TFR strategy for the youngers and to use the more potent 2nd generation TKIs first-line, in order to obtain the DMR. However, the indications for the TFR strategy have been recently re-discussed and the use of the second generation TKIs has been considered not cost-ineffective [43]. In light of the latter considerations, pursuing the TFR strategy in all CML patients, regardless of age and type of TKI, appears questionable. In almost all diseases, the age is one of the most important factors when care must be provided and goals are often very different in the youngers and in the elderly. The reasons why different age-related strategies are not considered in the case of CML need to be further discussed.

Other strategies rather than TD have been investigated during the last years: this is the case of intermittent TKI therapy. This strategy has been carried out in two Italian prospective multicenter trials: the INTERIM phase II and the ongoing OPTkIMA phase III trials [44,45,46]. In the first study, IM was given intermittently [44,45], one month on and one month off, in elderly (>65 years) patients with long lasting (>2 years) MR3.0/MR4.0. In the second study, IM, NIL or DAS are being progressively de-escalated in a randomized phase III trial (fixed arm: one month on and one month off versus progressive arm: one month on and one month off for the 1st year, one month on and two months off for the 2nd year, one month on and three months off for the 3rd year) [46].

The aim of these studies is to maintain at least the MMR (MR3.0) with a decreased TKI dose (50% less or more), possibly reducing the long-term toxicity and improving the quality of life (QoL). The first experience on the intermittent IM administration (INTERIM) showed that 60% of patients remain on trial and in MMR (MR3.0) after 7 years of follow-up, with saved costs and toxicity [44].

Many physicians consider this approach a rearguard strategy but, since the large majority (>60–70%) of CML patient are excluded from TFR, a policy based on the use of the minimal effective dose to maintain at least the MMR should be considered a pragmatic and sustainable treatment option.

More recently, Clark et al. indicated that a 1-year de-escalation of treatment (IM 200 mg/day) could be a useful strategy for a better selection of the candidates to the following TD [47]. This “clinical” selection based on the maintenance of MR at the 12th month may be improved by monitoring strictly the slope of the MR and by selecting for TD those patients with a MR slope showing a stability of BCR-ABL1 transcript levels.

Despite all the strategies mentioned above, it is crucial to remember that the main goal in CML is still to avoid potential progressions to advanced phases. Failure of second generation TKIs is a quite common risky situation. A recent study showed that 13% of patients treated with second-generation TKIs first line in clinical practice discontinue treatment. Similarly to data in second line, the most common causes of failure are resistance and intolerance [48]. The last update of ELN recommendations are in favor of the use of ponatinib in cases of resistance to second generation TKIs due to a presumably higher response rate compare to bosutinib and other second generation TKIs [20]. When considering treatment options in this situation, is it vital to take into consideration the probabilities of suffering from potential severe side effects, in order to avoid them. The use of ponatinib in high cardiovascular risk patients is a challenging situation. Ponatinib dose reductions in patients achieving response have been related to less side effects. Nevertheless, at present, the best starting dose, or the benefits of concomitant antiaggregants, remain uncertain [49]. New TKIs are being tested in this group of patients. Asciminib is a new BCR-ABL1 inhibitor that differs from previous TKIs approved in CML in that it does not bind to the ATP-binding site of the Kinase. Recent published data showed high rates of patients achieving optimal responses, with a favourable safety profile in previous heavily pre-treated populations [50]. Of interest, this compound is the first TKI that can be combined with “classical TKIs”, which could be of interest in patients harboring different resistant mutations, as well as to prevent the outgrowth of novel ones. Data from ongoing clinical trials will determine whether asciminib will be a new treatment option to manage our patients.

In all patients who fail therapy, BCR-ABL1 kinase domain mutation profiling is recommended since it may provide an important piece of information that complements safety considerations in the therapeutic decision algorithm [51]. BCR-ABL1 kinase domain mutations are not the only mechanism that may trigger TKI resistance but they are the only actionable one, since each first and second-generation TKI is known to have a precise spectrum of resistant mutations—and ponatinib might display reduced efficacy against some compound mutations [52]. A series of retrospective studies and a recent large prospective study have shown that the use of next generation sequencing (NGS) facilitates sensitive detection and quantitative assessment of mutations in patients with ’Failure’ and ’Warning’ responses [53,54,55,56,57]. NGS may identify and quantitate mutations in BCR-ABL1 transcripts down to 1%–3% abundance and resolve the clonal architecture in the majority of cases harboring multiple mutations, thus easily discriminating between compound mutations (two mutations in *cis*) and polyclonality (two mutations in *trans*) [58]. The ELN panel has incorporated the use of NGS in the latest treatment recommendations [20].

What about the perspectives?

In the future, CML therapy will have to face the eradication of Ph+ LSC. At present, none of the TKIs seems to be able to achieve this result, neither through a direct drug action nor through a time-dependent mechanism sustained by continuous drug pressure and/or immune surveillance effects leading to a progressive exhaustion of Ph+ LSC. Immunologic mechanisms are often invoked but, at present, they remain hypothetical [59,60,61]. Some evidence on a specific role of lymphocyte sub populations (NK) has been reported, but deeper studies of the immunologic mechanisms in CML patients during TKI treatment should be addressed [62,63]. Combinations of TKIs with other drugs having different mechanisms of action should be tested, even if no new effective molecules with this potential are currently available and, in any case, the combinations tested “in vitro” must be safe “in vivo”.

The current and the future CML therapy with TKIs would be really personalized and adapted to the risk of the disease, the patients’ age, the TKIs’ potency and toxic profile, and the latency of the molecular response.

Probably, proposing the TFR strategy to all patients by using any type of TKI is not recommendable. As with the risk of disease, we believe that therapy and the goals of treatment should be different and age-related. In the younger CML patients, it could be reasonable to pursue a TFR strategy by employing upfront the more potent TKIs, aiming to quickly achieve a DMR for discontinuing the treatment earlier. In the older patients, it could be reasonable to adopt a strategy of the minimal effective dose to save toxicity and drug costs, and to improve the tolerance and QoL [64].

Having a precise and accurate methods for MR measurement is mandatory to better design the future strategies, to optimize the CML therapy and to refine patient management, but it is difficult to properly manage CML therapy when RT-qPCR provides results of MRD expressed as “undetectable transcript” [32]. Digital PCR (dPCR) may overcome this intrinsic limit of RT-qPCR and is the only feasible tool in the event of stopping TKIs for a better selection of CML patients eligible to treatment discontinuation. By now, retrospective and prospective data [33,65,66,67] clearly confirm the high sensitivity and accuracy [68,69,70] of dPCR for assessing the MRD in CML cases presenting undetectable MRD by RT-qPCR and reinforce the evidences of dPCR capability in predicting TFR, after TKIs discontinuation. The positive predictive value for dPCR ranges between 68 and 87% [33,66].

These considerations strongly support the usefulness of dPCR as the unique feasible alternative to RT-qPCR to provide a robust, sensitive and accurate quantitation of BCR-ABL1 in routine clinical practice. In the era of more potent TKIs, precision medicine and personalized treatment programs, it is the time to introduce the dPCR in the management of the future CML therapy. Having an accurate and precise method is essential if the eradication of the disease has to be measured. Additionally, as a relatively simple and cost-effective approach allowing to obtain rapid results with no need of dedicated bioinformatic skills, dPCR might in the future prove complementary to, or even compete with NGS, at least for the detection of critical BCR-ABL1 mutations like the pan-resistant T315I.
